# Ligand Activation of the Prokaryotic Pentameric Ligand-Gated Ion Channel ELIC

**DOI:** 10.1371/journal.pbio.1001101

**Published:** 2011-06-21

**Authors:** Iwan Zimmermann, Raimund Dutzler

**Affiliations:** Department of Biochemistry, University of Zurich, Zurich, Switzerland; Harvard Medical School, United States of America

## Abstract

While the pentameric ligand-gated ion channel ELIC has recently provided first insight into the architecture of the family at high resolution, its detailed investigation was so far prevented by the fact that activating ligands were unknown. Here we describe a study on the functional characterization of ELIC by electrophysiology and X-ray crystallography. ELIC is activated by a class of primary amines that include the neurotransmitter GABA at high micro- to millimolar concentrations. The ligands bind to a conserved site and evoke currents that slowly desensitize over time. The protein forms cation selective channels with properties that resemble the nicotinic acetylcholine receptor. The high single channel conductance and the comparably simple functional behavior make ELIC an attractive model system to study general mechanisms of ion conduction and gating in this important family of neurotransmitter receptors.

## Author Summary

Electrical signal transduction at chemical synapses of nerve and muscle cells is initiated by the binding of neurotransmitters to their receptors, which function as gated ion channels that open a selective ion conduction path in the ligand-bound state. The pentameric ligand-gated ion channels constitute a very important family of neurotransmitter receptors that includes, amongst others, the nicotinic acetylcholine receptor. While most pLGICs are expressed in higher eukaryotes, there are few family members found in bacteria. These bacterial channels share the overall structural features of the family and are thus expected to function by similar mechanisms, but although the channel from the bacteria *Erwinia chrysanthemi* has revealed first structural insight into the family at high resolution, its functional characterization was impossible since activating ligands were not known. In our study we have identified ligands activating ELIC and we have located their binding by X-ray crystallography. Ligand binding causes the transition into an open state, which, as in eukaryotic receptors, is only transient and which slowly inactivates in the presence of the ligand. By single channel analysis we found that, similar to the acetylcholine receptor, ELIC forms cation selective channels. With known ligands ELIC has become an important model system to study the general mechanisms of the family.

## Introduction

The pentameric ligand-gated ion channels (pLGICs) constitute a large family of ionotropic neurotransmitter receptors that are ubiquitously expressed in the animal kingdom. In vertebrates the family encompasses cation selective serotonin and nicotinic acetylcholine receptors (nAChRs) and anion selective GABA and glycine receptors [1]. All family members share a conserved molecular architecture and a similar functional behavior. The proteins act as gated ion channels that are closed in their resting state and that open a selective ion conduction pore upon the binding of a ligand to a site located on an extracellular domain [2]–[6]. These properties are shared by close prokaryotic homologues that are encoded in the genome of certain bacterial species [7]–[9]. The X-ray structures of two prokaryotic pLGICs from the plant pathogen *Erwinia chrysanthemi* (or *Dickeya dadantii*, ELIC) [10] and the cyanobacterium *Gloebacter violaceous* (GLIC) [11],[12] have recently revealed first structural insight into the family at high resolution. While both structures are similar overall, distinct conformational differences in the ligand binding domain and the transmembrane pore domain indicate that they represent different functional states of the protein, with ELIC showing a non-conducting and GLIC a conducting state (Figure S1).

The functional properties of GLIC, which is opened by a decrease of the extracellular pH, have been investigated by electrophysiology [13]. The protein forms cation selective channels with slow opening kinetics and currents that are stable over an extended period of time. The latter property differs from most eukaryotic pLGICs that quickly inactivate after opening [14]–[16]. The analogous ion permeation properties and the sensitivity to the same set of open channel blockers underline the close structural relationship of the ion conduction pore between GLIC and the nAChR [13],[17], which is also manifested in the sequence conservation of this region (Figure S1G). Although, based on sequence similarity and an initial functional characterization [10], ELIC is also expected to belong to the same group of cation selective channels, it has so far been impossible to investigate its detailed properties since the stimulus for its activation was unknown.

**Figure 1:**
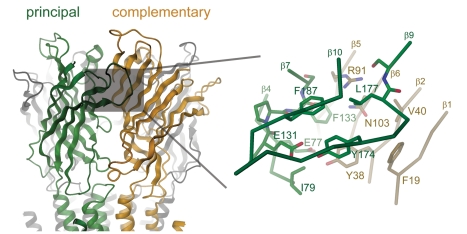
Ligand binding region of ELIC. Left: ribbon representation of the ligand binding domain of ELIC. The subunits contributing to the principal and complementary side of the binding region are colored in green and orange, respectively. The binding site is marked by a grey box. Right: zoom into the binding site. The protein is shown as Cα-trace with residues lining the binding pocket shown as stick model. The residues and secondary structure elements are labeled. Structures displayed in Figures 1, 3, and 5 were prepared with DINO (www.dino3d.org).

ELIC shows a non-conducting conformation of the pore [10],[18], whose correspondence to the resting state of eukaryotic pLGICs is currently unclear [7],[19]. The structural similarity of its extracellular part to the acetycholine binding protein (AChBP), a soluble protein that closely resembles the ligand-binding domain of nAChRs, however, suggests a conserved mode for ligand interactions (Figure 1, Figure S2) [10],[20]. Structures of the AChBP in complex with different agonists of the nAChR have provided a detailed picture of this process and have helped to rationalize previous biochemical studies [3],[21]–[23]. The ligand binding site is placed in a pocket at the interface of adjacent subunits of the pentameric protein. The two halves of the pocket are termed “principal” and “complementary side,” respectively, depending on the subunit in which the contributing residues are located. On each subunit residues from different regions of the molecule line the ligand binding pocket. Their location was named according to early experiments (Figure S2A) [24]. Based on this nomenclature the principal side contains the regions A, B, and C and the complementary site the regions D, E, and F. The conserved residues include several aromatic side chains, which provide delocalized electrons to stabilize the positively charged quaternary ammonium group (Figure S2C) [25],[26]. The structural similarity between ELIC and AChBP extends to the ligand binding site, which also in ELIC contains conserved aromatic positions that surround a pocket of about the same volume (Figure 1, Figure S2). It was thus assumed that ELIC might be opened by a ligand of similar size and chemical properties [10].

Here we have investigated the activation of ELIC by small molecular ligands by X-ray crystallography and different electrophysiological techniques. Our study has identified a class of primary amines that open the channel by a strongly cooperative process. Although the open state is comparably stable the channels desensitize with slow kinetics. An X-ray structure of ELIC in complex with a ligand shows electron density in the consensus binding site, thus suggesting that the mechanism of activation is conserved. The open channel of ELIC mediates the permeation of cations with a large single channel conductance and selectivity properties that resemble other cation selective members of the family.

## Results

### Activation of ELIC

**Figure 2:**
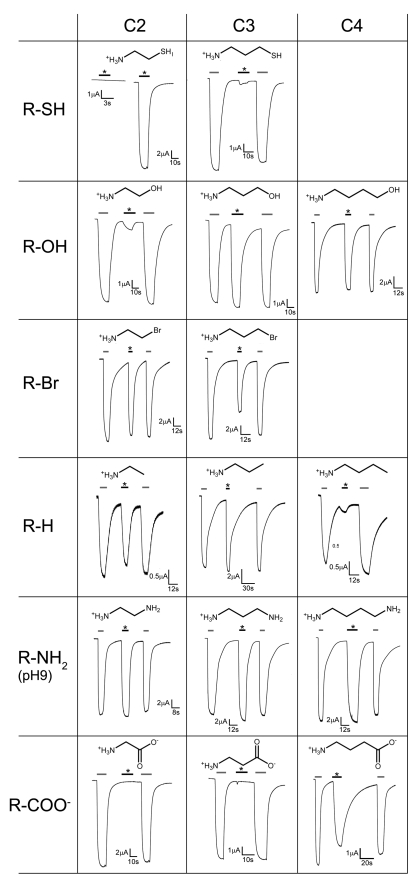
Current response of ELIC to different ligands. The chemical structures of the ligands are shown above the traces. Currents were recorded from oocytes at −60 mV with the two electrode voltage clamp technique. Top left: the response to 2 mM cysteamine is shown for water-injected control oocytes and for oocytes expressing ELIC. The application of the ligand is indicated (bar, *). All other traces show the response to agonists in comparison to the response to cysteamine. Agonists were applied to the outside in a concentration of 10 mM. The activation by cysteamine (grey bar, 2 mM) is followed by the application of a different agonist (black bar, *, 10 mM) and another activation by cysteamine (grey bar, 2 mM). Ligands are grouped according to their chemical properties and length of the aliphatic chains.

To identify ligands that activate ELIC we have expressed the protein in *Xenopus laevis* oocytes and investigated the response to potential agonists by the two-electrode voltage-clamp technique. Based on the volume and chemical properties of the proposed ligand binding region and the presence of positively charged ions bound to this site, we reasoned that parts of the agonists might have a cationic character [10]. We have thus selected a set of cationic and zwitterionic compounds with an approximate size of neurotransmitters for screening. Our library included different natural products such as amino acids and their derivatives, metabolites, and related synthetic substances (Figure S3A). Among the investigated molecules, only those containing a primary amino moiety at one end of a linear two- to four-atom-long carbon chain showed an activating response on ELIC-mRNA injected oocytes when added to the extracellular solution in low millimolar concentrations (Figure 2). The same compounds did not have any effect on water-injected control oocytes. Besides alkylamines all ligands contain atoms with lone electron pairs on the other end of the carbon chain. The size of the agonist is dependent on its chemical nature. For amino alcohols strong activation is obtained for amino-butanol and amino-propanol (Figure 2). The response to brominated amines is shifted towards shorter chain-lengths, with bromoethylamine and bromopropylamine both opening the channel. Ethylamine and propylamine activate the channel while the effects of butylamine and the branched isopropylamine are weak (Figure 2, Figure S3B). Among aminothiols, cysteamine is the most efficacious, while the response to homocysteamine is small. Different polyamines from diaminoethane to putrescine, but not cadaverine, activate ELIC in a state where one of the two amino groups is deprotonated. Due to the shift of one of its pK_a_ values, the short diamine aminoethane is thus already active at neutral conditions, while a significant effect by the natural product putrescine is only observed at higher pH (Figure 2, Figure S3B). Remarkably also the neurotransmitter GABA shows an activating response at low mM concentrations, while the response to the shorter beta-alanine and glycine is absent (Figure 2).

**Figure 3:**
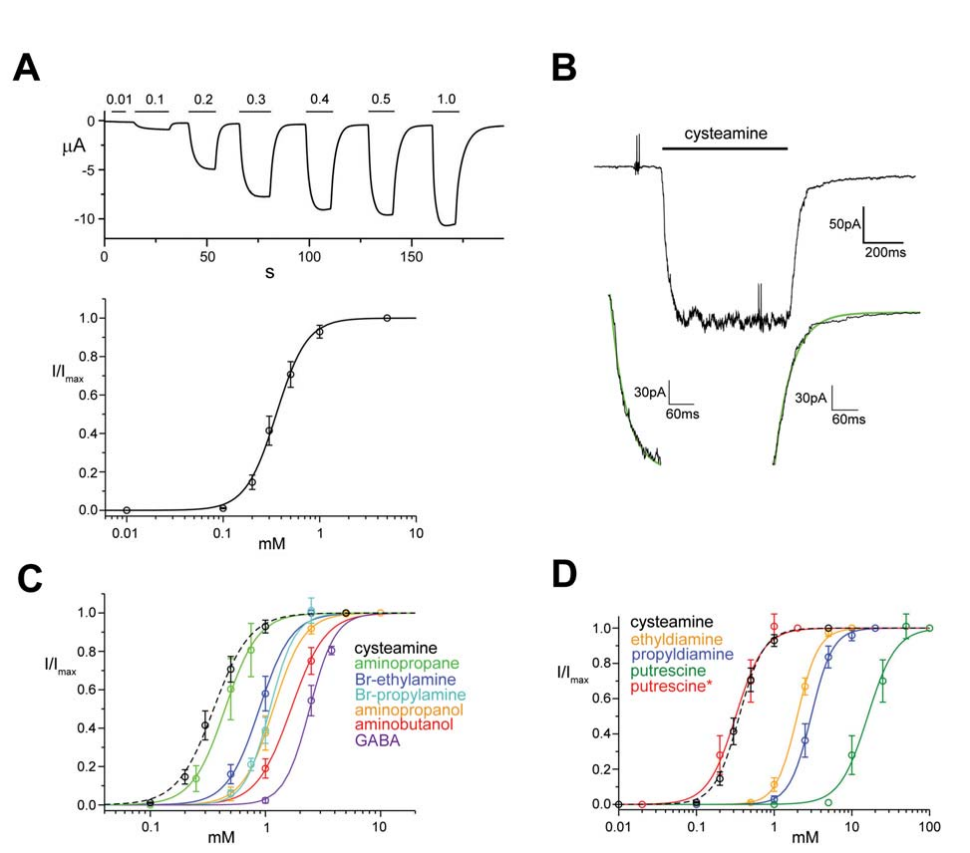
Ligand activation of ELIC. (A) The current response upon application and washout of cysteamine was recorded at −60 mV with the two-electrode voltage clamp technique. The application of cysteamine at the respective concentration is indicated above (black bar). The relative open probability (I/I_max_) plotted as a function of the ligand concentration is shown below. The currents were normalized to the maximum at saturating ligand concentration (i.e., 5 mM). The average of 10 oocytes and their standard deviations are shown. The solid line shows a fit to a Hill equation with a coefficient of 2.7. (B) Activation and deactivation kinetics. Macroscopic currents from a membrane patch in the outside-out configuration were recorded at −60 mV in response to a fast exchange into solutions containing 5 mM cysteamine. The fit of the current increase upon application of the ligand and the decrease upon washout to a single exponential function is shown below. (C) Dose response curves for the activation of ELIC by different agonists. Currents were recorded with the two electrode voltage clamp technique at −60 mV. The solid lines show fits to a Hill equation. The dose response curve for cysteamine (black, dashed line) is shown for comparison. (D) Dose response curves for the activation of ELIC by diamines. Currents were recorded with the two electrode voltage clamp technique at −60 mV and pH 8. The solid lines show fits to a Hill equation. The red trace (putrescine*) shows a fit to the fraction of putrescine at pH 8 that carries a single positive charge. The dose response curve for cysteamine (black, dashed line, measured at pH 7) is shown for comparison.

The strongest response at low ligand concentration is evoked by cysteamine, the decarboxylation product of the amino acid cysteine. We have thus used this molecule as an agonist in most subsequent experiments. At the pH of the recording solution, the majority (>96%) of the sulfhydryl groups (pK_a_ = 8.4) are protonated and ligands thus carry a positive net-charge. Stable currents over a period of seconds were obtained upon addition of high micromolar concentrations of cysteamine to the extracellular medium (Figure 3A). At longer incubations with the ligand, however, the currents decayed, which is indicative for a slow desensitization of the channel (Figure S4A). The channel shows a steep voltage-independent activation with an EC_50_ of 365 µM and a Hill coefficient of 2.7, which points at a strongly cooperative process. To follow the time-course of activation and deactivation, we have investigated excised outside-out patches in response to fast solution exchange. A similar phenotype of currents as previously seen in two-electrode voltage clamp recordings, although with faster kinetics of desensitization, was also observed for macroscopic currents in a patch clamp experiment (Figure 3B, Figure S4B). At 5 mM cysteamine, channel activation and deactivation can be approximated by exponential functions with time constants in the ms range (τ_act_ = 20±5 ms, *n* = 19, τ_deact_ = 53±7 ms, *n* = 19, all errors are s.d.). As expected for ligand-gated channels, the activation but not the deactivation rates are dependent on the ligand concentration (Figure S4C). Since the observed time constants are within the range required for solution exchange, the values are upper limits for the processes and might even proceed with somewhat faster kinetics.

Other agonists activate ELIC in a similar cooperative process as cysteamine, although at higher concentrations (Figure 3C). A ranking of the ligands with respect to their potency is shown in Table 1. In most investigated cases, the responses at saturating ligand concentrations were comparable to cysteamine, thus suggesting that the corresponding maximum open probabilities of the channel are close (Figure 2, Table 1). GABA opens the channel with an EC_50_ of 2.4 mM, which is higher than that observed for other ligands. At a fixed pH, the potency of diaminoalkanes decreases with increasing chain length, partly reflecting the protonation state of the molecule. Although at pH 8.0 the EC_50_ of putrescine is high, it significantly decreases at higher pH values and it is similar to cysteamine if only the fraction of molecules is considered where one of the two amino groups is uncharged (Figure 3D).

**Table 1:** Dose-response relationships of different agonists of ELIC.

	EC_50_/mM	n	pH_EC50_	I_max_/I_max_^cysteamine^ *	pH_I/I_
cysteamine	0.365	2.7	7.0	1	7.0
aminopropane	0.446	3.0	7.0	0.99±0.02	7.0
bromoethylamine	0.885	3.1	7.0	0.94±0.04	7.0
bromopropylamine	1.1	3.9	7.0	0.71±0.03	7.0
aminopropanol	1.2	3.2	7.0	1.00±0.014	7.0
aminobutanol	1.7	2.9	7.0	0.98±0.03	7.0
GABA	2.4	3.9	7.0	0.79±0.09	7.0
diaminoethane	2.0	3.2	8.0	1.07±0.06	9.0
diaminopropane	3.0	3.1	8.0	1.08±0.03	9.0
putrescine	16.0	2.4	8.0	1.03±0.07	9.0
putrescine+	0.23	2.4	8.0		

**+:** concentration of monovalent putrescine.

*Averages and s.d. of six measurements.

### The Ligand Binding Pocket

The chemical nature of the activating ligands as short unbranched primary amines and the response of ELIC to the neurotransmitter GABA mirror the structural conservation in the ligand binding pocket (Figure 1, Figure S5). In its center the binding site is framed by aromatic residues that are found in all members of the family, but among different pLGICs the relationship is closest to inhibitory neurotransmitter receptors such as GABA and glycine receptors [2],[27]–[29]. It is thus noteworthy that the gene encoding for ELIC (protein_id: ADN00343.1) has been annotated as a GABA receptor homologue. On the principal side the conserved residues include Glu131 and Phe133 on β-7 (loopB), Tyr 174 on β-9, and Phe187 on β-10 (loopC). On the complementary side, Tyr38 on β-2 (loop D) and Asn103 on β-6 are conserved. The differences in other residues lining the pocket likely account for the unique agonist specificity of ELIC when compared to eukaryotic receptors.

**Figure 4:**
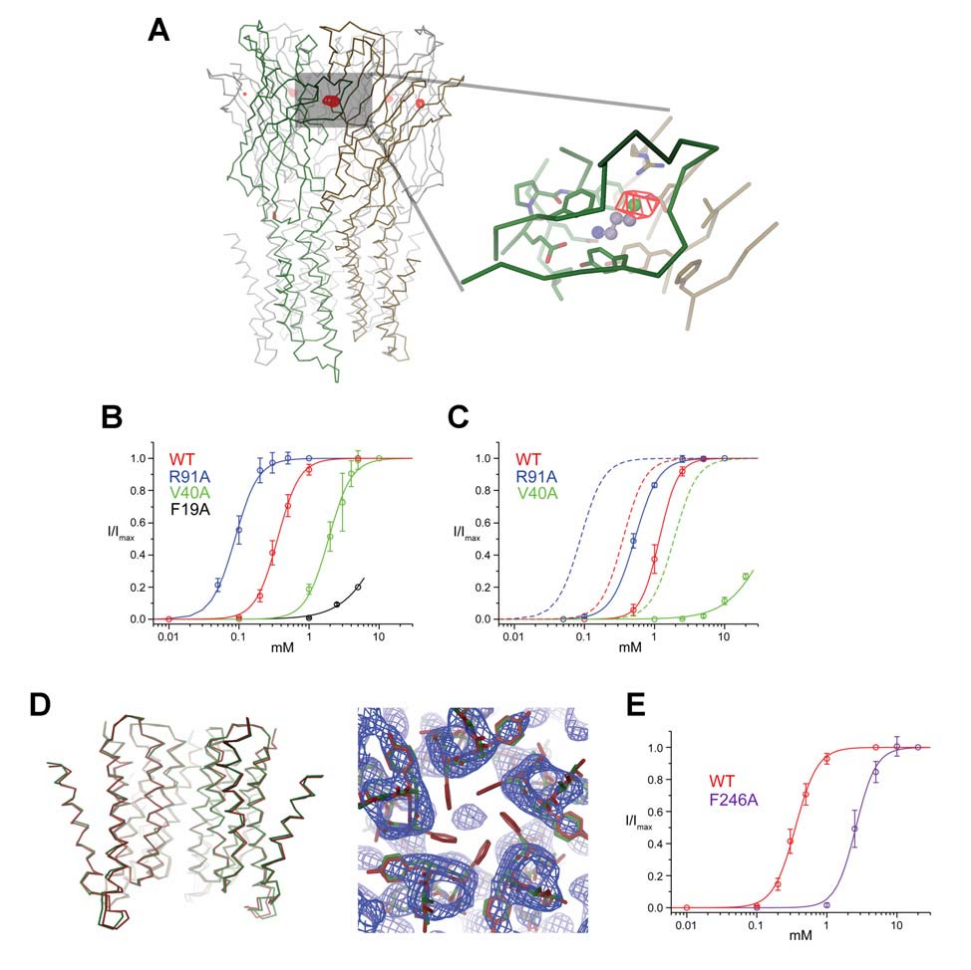
Ligand binding and mutagenesis. (A) Anomalous difference map of ELIC in complex with bromopropylamine. One of two pentamers of ELIC in the asymmetric unit of the crystal and a zoom into a single binding pocket are shown. The subunits of the principal and complementary side are colored in green and orange, respectively. The anomalous difference map calculated at 5.0 Å and contoured at 4.5 σ is shown as red mesh. The ligand binding region is indicated by a grey box. A model of bromopropylamine is shown in ball and stick representation. (B) Activation of ligand binding site mutants by cysteamine. Dose response curves from currents recorded with the two-electrode voltage clamp technique are shown. The solid lines show fits to a Hill equation. (C) Activation of ligand binding site mutants by aminopropanol. The solid lines show fits to a Hill equation, and dashed lines in the same color show the activation of the respective mutants by cysteamine. (D) Structure of the mutant F246A. Left: Superposition of Cα traces of the pore region of WT (red) and the mutant F246A (green). The view is from within the membrane; the front subunit is removed for clarity. Right: 2Fo-Fc electron density (calculated at 3.3 Å and contoured at 1 σ, blue mesh) of the pore region of the mutant F246A superimposed on the refined structure (green). The WT structure (red is shown for comparison). The view is from the extracellular side. The missing electron density for the aromatic side chains is apparent. (E) Activation of the pore mutant F246A by cysteamine. Dose response curves from currents recorded with the two-electrode voltage clamp technique are shown. The solid lines show fits to a Hill equation. The WT is shown for comparison.

In an attempt to locate bound agonists we have soaked bromopropylamine into crystals of ELIC and we have collected data at the anomalous absorption edge of bromine. The overall structure of the protein was not visibly affected by this procedure. Despite the comparably low resolution of the data, strong peaks in anomalous difference Fourier maps in all binding pockets of the two the channels in the asymmetric unit reveal the presence of bound ligands (Figure 4A). When placing bromopropylamine into these sites the primary amino group is located close to the two carboxyl groups of Glu77 and Glu131 of the principal subunit and the bromide moiety is found close to the residues Asn103 and Arg91 at the complementary side.

To investigate the role of residues surrounding the bound ligand for channel activation, we have mutated single residues to alanine and we have measured activation with the two-electrode voltage-clamp technique (Table 2). For many mutants there was no response to the addition of cysteamine. This was the case for all investigated residues of the principal site, such as Glu77 and Glu131, that may interact with the primary amino group and hydrophobic and aromatic residues (i.e., Phe187, Phe133, Tyr174, Ile79) that surround the aliphatic carbon atoms of the ligand. The effect of mutations on the complementary side, in contrast, was diverse and in some cases less drastic. The mutations of Phe19 on β-1 and Val40 on β-2 shifted the activation towards higher agonist concentrations (Figure 4B). The mutations of two residues that were found close to the bromine position in the crystal structure had opposite effects: While the mutation of Asn103 did not show an activating response, the truncation of a nearby Arg91 increased the apparent affinity for the ligand as reflected in a 4-fold decrease of the EC_50_ for cysteamine and a smaller 2-fold decrease for aminopropanol (Figure 4B,C). The detection of bound ligands in an X-ray structure and the strong effect of mutations in the same region emphasize the role of the ligand binding site for channel activation and demonstrate that the general agonist-binding mode that has been thoroughly characterized for the AchBP is also conserved in ELIC.

**Table 2:** Dose-response relationship of activation of ELIC point mutants.

	cysteamine		aminopropanol	
	EC_50_/mM	n	EC_50_/mM	n
WT	0.365	2.7	1.2	3.2
R91A	0.087	2.6	0.511	2.45
V40A	1.9	2.6	>10	
F19A	>10		—	
F246A	2.59	3.0	n.d.	

### The Role of Aromatic Pore Residues for Channel Activation

In contrast to the extracellular domain, the pore of ELIC resembles cation selective members of the family that include nAChRs, serotonin receptors, and the prokaryotic channel GLIC (Figure S1G). In cation selective pLGICs, three layers of hydrophobic side-chains provide an overall apolar character to the extracellular half of the pore region while the following two layers of polar residues and a layer of negatively charged residues render the intracellular half hydrophilic [7],[30]. In a recent study, it was suggested that interactions between aromatic residues (i.e., Phe246) of the outer hydrophobic layer would stabilize the closed conformation of ELIC [31]. To investigate the role of this aromatic group for channel opening, we have mutated it to alanine and studied the mutant by X-ray crystallography and electrophysiology. The structure of the mutant F246A was determined at 3.3 Å resolution and shows a conformation that is, apart from the side chain truncation, virtually identical to WT (Figure 4D, Table 3). In electrophysiological recordings we found that the EC_50_ of cysteamine activation of F246A was shifted towards higher concentrations (Figure 4E). This shift is due to a mutation of a residue located remote from the ligand binding site and thus likely reflects a decreased efficacy of the channel. Our results thus do not provide any evidence that the ring of phenylalanine residues, which is unique to ELIC, would stabilize the pore in an unusual non-conducting conformation.

**Table 3:** Data collection and refinement statistics.

	ELIC 5 mM bromopropylamine	ELIC F246A
**Data collection**		
Space group	P2_1_	P2_1_
Cell dimensions		
*a*, *b*, *c* (Å)	105.2, 266.7, 110.6	105.4, 266.2, 110.9
α, β, γ (°)	90.0, 110.1, 90.0	90.0, 110.7, 90.0
Resolution (Å)	40–4.0	40–3.3
*R*_merge_ *	9.2 (85.2)	7.1 (76.4)
*I*/σ*I* *	9.8 (1.8)	15.1 (2.0)
Completeness (%)*	99.5 (99.4)	99.0 (95.9)
Redundancy*	3.5	3.5
**Refinement**		
Resolution (Å)	20–4.0	15–3.3
Number of reflections	48,130	83,739
*R*_work_/*R*_free_	29.5/29.5	23.9/26.1
Number of atoms		
Protein	n/a	24,960
R.m.s. deviations		
Bond lengths (Å)	n/a	0.01
Bond angles (°)	n/a	1.5

*Values in parentheses are for highest resolution shell.

### The Conduction Properties of the Ion Channel Pore

**Figure 5:**
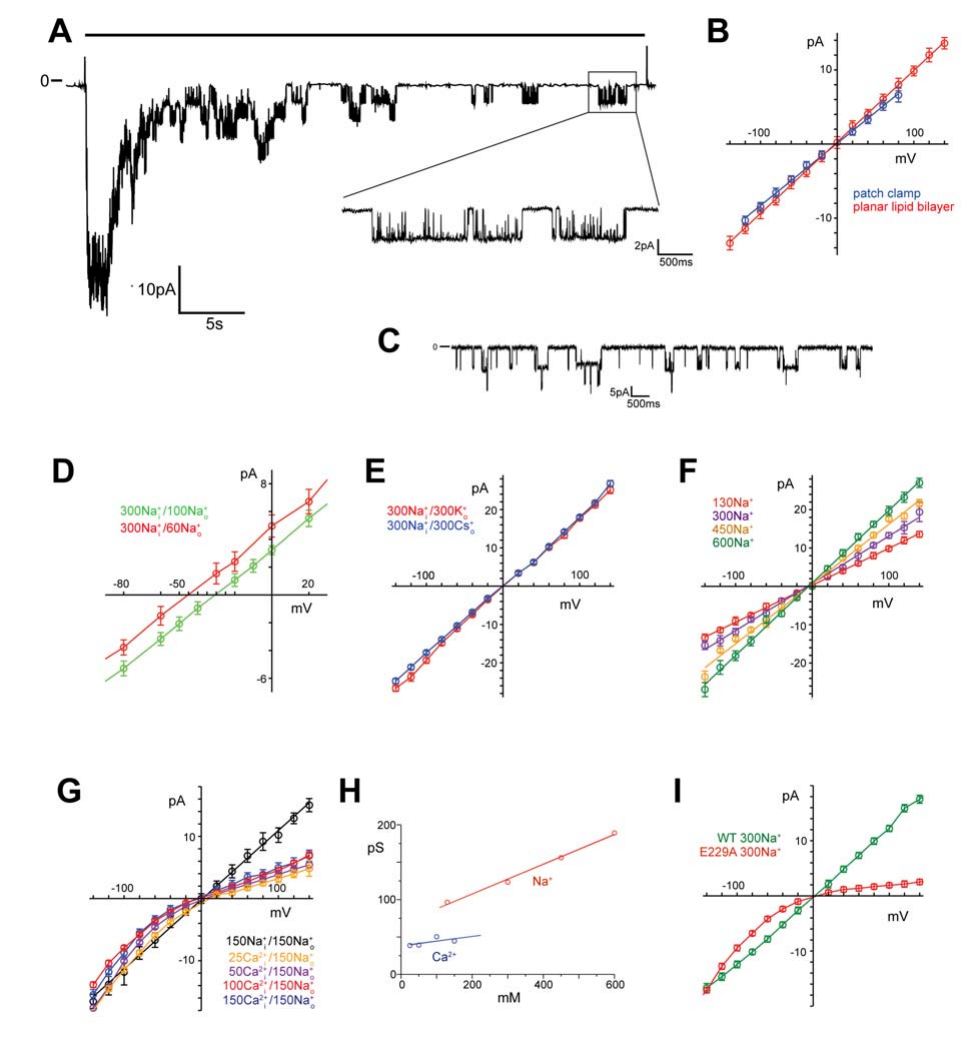
Single channel recording of ELIC. (A) Patch clamp recordings of ELIC expressed in *Xenopus oocytes* in the outside-out configuration at −60 mV. The agonist (5 mM cysteamine) was applied by a fast solution exchange (black bar above). The decrease of channel activity over time is due to desensitization. The magnified trace shows activity of a single channel. Recordings were filtered at 1 kHz. (B) Current-voltage relationships recorded from single channels of ELIC expressed in *Xenopus oocytes* in a patch clamp experiment (blue) and of reconstituted protein in artificial lipid bilayers (red). The errors are standard deviations (s.d.) obtained from fits to current amplitude histograms. (C) Current trace from a planar lipid bilayer containing at least two channels recorded at −100 mV in the presence of 2 mM cysteamine (recordings were filtered at 200 Hz). (D) Current voltage relationships of ELIC in asymmetric concentrations of NaCl measured in the presence of 2 mM cysteamine. The currents reverse at the Nernst potential of Na^+^. The compartment with lower ion concentration corresponds to the “extracellular side.” Data shown in panels D–G and I were measured from single channels in planar lipid bilayers. The errors are s.d. obtained from fits to current amplitude histograms. Channels were activated by addition of 2 mM cysteamine to both compartments unless stated differently. (E) Current voltage relationships in asymmetric conditions containing equivalent amounts of different monovalent salts. (F) Current voltage relationships of single channel currents at different concentrations of NaCl. (G) Current voltage relationships in asymmetric salt conditions. The “extracellular side” contains 150 mM NaCl, and the “intracellular side” contains different concentrations of CaCl_2_ (25–150 mM). Channels were activated by addition of 2 mM cysteamine to the “extracellular side” only. Data from symmetric concentrations of NaCl (150 mM, black) are shown for comparison. (H) Concentration dependence of the single channel conductance of Na^+^ and Ca^2+^ currents. (I) Current-voltage relationships of the mutant E229A in symmetric concentrations of NaCl. Channels were activated by addition of 2 mM cysteamine to the “extracellular side.” Data from the WT channel (green) are shown for comparison.

To characterize the ion conduction properties of ELIC, we have investigated single channel currents by patch-clamp and by fusion of reconstituted protein to artificial lipid bilayers (Figure 5, Figure S6). In recordings of patches excised from *Xenopus* oocytes expressing low levels of ELIC in the outside-out configuration, the opening of single channels upon addition of cysteamine can be observed (Figure 5A). These channels show an ohmic behavior in a broad voltage range with a single channel conductance (g) of 84 pS (Figure 5B). At high ligand concentrations, the initially strong channel activity decreases over time due to the desensitization of the channel. At prolonged incubation with the ligand, thus, bursts of activity alternate with long closures (Figure 5A). Within the bursts the channels are predominantly in an open state that is only interrupted by brief closures, which is consistent with a high efficacy of the ligand [32]. The openings recorded from ELIC, which was overexpressed and purified from *E. coli* and reconstituted into planar lipid bilayers, show a channel with very similar behavior (Figure 5C). Channel activity is evoked upon addition of the ligand to either side of the bilayer, thus indicating that the reconstituted channels are oriented in both ways. As for patch clamp recordings also in bilayers, long periods of closures are interrupted by burst of openings. In bilayers, and less frequently in patches, we have also observed the occasional transition of the open channel to a subconductance of about 80% of the current amplitude (Figure S6A). A similar subconductance was previously described for the nAChR [33]. At corresponding ion concentrations the conduction properties of reconstituted ELIC in planar lipid bilayers are very similar as observed in patch clamp recordings of the channel expressed in *Xenopus laevis* oocytes (for the main conductance level g = 96 pS), thus emphasizing that the functional behavior of the protein is largely independent of its expression host (Figure 5B). In gradients of NaCl, single channel currents reverse at the Nernst potential of Na^+^, thus indicating that the channel is cation selective and the permeability to anions is negligible (Figure 5D). Under asymmetric conditions with the same concentrations of different monovalent salts on either side of the membrane, the currents reverse at 0 mV, showing that there is no measurable discrimination between monovalent cations (Figure 5E). This functional behavior resembles the ion selectivity of the nAChR [34],[35] and it matches the behavior of the protein described for macroscopic currents obtained at high protein-to-lipid ratio in the absence of the ligand [10]. The single channel conductance linearly increases with the ion concentration and does not show any saturation at high concentrations (Figure 5F). To characterize the conduction behavior of divalent ions, we have replaced the monovalent cations at the “intracellular side” with different concentrations of divalent cations and we have activated the channel by addition of agonists to the “extracellular side.” The IV curve in this case shows weak rectification with a lower slope conductance at positive voltages where divalent ions permeate the channel from the intracellular side (Figure 5G). This behavior is observed for different divalent cations such as Ca^2+^ and Ba^2+^ (Figure S6C). In contrast to monovalent ions, the concentration dependence of the Ca^2+^ conductance is shallow and nearly saturates already at low ion concentration (Figure 5H). This property was previously observed in nAchRs, where divalent ions in general interact stronger with the channel than their monovalent counterparts [36]–[38]. In all cation selective members of the family, a conserved negatively charged residue at the intracellular pore entry termed the “intermediate ring of charge” plays an important role for ion conductance, selectivity, and the interaction with divalent ions (Figure S1G) [39],[40]. In the mutant E229A, the charge corresponding to this ring of residues is removed. When investigated in planar lipid bilayers after addition of ligand to the “extracellular side,” a large effect on ion conductance is apparent. The currents show pronounced rectification with a strong decrease of the conductance at positive voltages (Figure 5I). The mutant is less selective and also permits the conduction of small anions. This behavior is manifested in recordings from solutions where chloride is replaced by the larger anion glucuronate, which for steric reasons does not permeate the channel. In such cases, the conductance of the mutant E229A is strongly decreased, whereas the WT protein is hardly affected (Figure S6D). The distinct permeation properties place ELIC within the cation selective branch of the family. The relative stability of the open state and the high single channel conductance additionally make it an attractive model for detailed biophysical investigations.

## Discussion

Although the prokaryotic pLGIC ELIC has provided the first structural insight into this family of ligand-gated ion channels at high resolution, its functional characterization was so far prevented by the fact that activating ligands were unknown. In this study we have identified agonists of ELIC from a library of molecules that were selected based on the chemical features of the ligand binding site, we have located a bound ligand in a crystal structure, and we have characterized the functional properties of the channel by electrophysiology.

**Figure 6:**
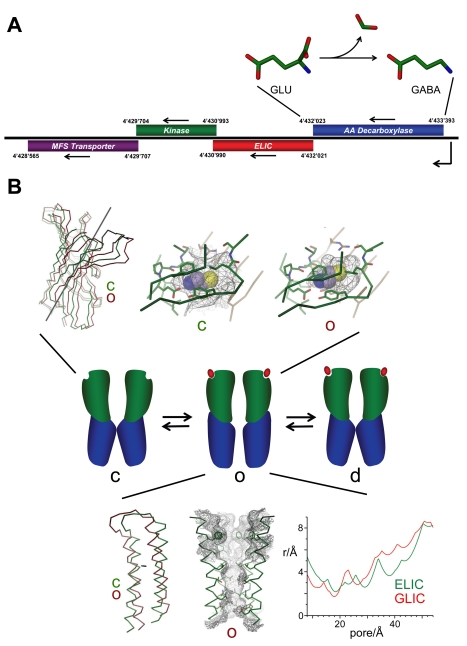
Biology and hypothetical mechanisms. (A) Localization of ELIC on the genome of *Erwinia chrysanthemi*. Open reading frames residing on the same operon and their respective positions on the gene are shown. The arrows indicate the direction of transcription. The annotation is based on sequence homology. The reaction catalyzed by the annotated glutamate decarboxylase (AA Decarboxylase) is shown above. (B) Potential conformational transitions during ligand activation. Upon ligand binding the channel changes from a resting state (c) to an open state (o) followed by a transition to a desensitized state (d). (Top) Comparison of the binding pocket of ELIC in a non-conducting conformation with a hypothetical structure of the region in a conducting state. The model of the conducting state (red) was generated by independent counterclockwise rigid-body rotations of the ligand binding domain of ELIC (green) by about 12° around the axis indicated in the figure. The rotation axis was obtained from a least square fit of conserved regions of the ligand binding domain of ELIC on the equivalent regions of GLIC. The binding sites of both states are shown. The ligand binding pockets are displayed as grey mesh. A model of cysteamine (shown as CPK representation) was placed into the binding site in a similar binding mode as observed in the structure of ELIC in complex with bromopropylamine. (Bottom) Hypothetical structure of the pore region in a conducting conformation. The conducting state of the pore region (red) was constructed by a rigid body rotation of the α2-α3 helix pair of a subunit of ELIC (green) by 12° in a counterclockwise direction around an axis that is indicated in the figure. The model of the pore region of this conformation is displayed as Cα trace. Side chains of pore forming helices are shown as sticks. The solvent accessible surface is shown as grey mesh. The front subunit is removed for clarity. The pore radii of GLIC (green, with the side chains of Glu 221 pointing away from the channel axis) and the hypothetical conducting conformation of ELIC as calculated with the program HOLE [61] are shown (the cytoplasm is on the left side).

ELIC is activated by a set of primary amines that include alkylamines, aminothiols, aminoalcohols, bromoamines, diamines, and the neurotransmitter GABA. Quaternary amines, such as acetylcholine, in contrast, are ineffective. The chemical properties of the agonists underline the conservation of the ligand binding pocket that is closer to GABA and glycine receptors than to nAChRs and AChBPs (Figure S5). As is the case for other pLGICs [41],[42], ligand activation in ELIC is a cooperative process and appears to involve the binding of at least three molecules to maximally activate the protein. However, unlike its eukaryotic counterparts [43], all agonists activate ELIC with comparably low apparent affinity at high µM to mM concentrations. While it is still not known whether the ligand activating ELIC in a biological context is among the characterized molecules, it is noteworthy that several of the agonists are breakdown products of amino acids, two of which, putrescine and GABA, may play a role in the natural environment of the bacteria, which as pathogens degrade the roots of plants. It may thus not be a coincidence to find the gene of a putative amino acid decarboxylase in the direct vicinity of the gene encoding for ELIC on the bacterial chromosome (Figure 6A). This protein, which might be involved in the processing of the ligand, was annotated as glutamate decarboxylase, thus suggesting that, despite its low potency, GABA is among the likely candidates for natural ligands.

The macroscopic opening and closing kinetics of ELIC is in the millisecond time range. Although the rates are slow compared to most eukaryotic family members [44],[45], they are similar to the previously investigated activation and deactivation rates of GLIC [13]. In contrast to most eukaryotic pLGICs [15], the evoked currents are stable for several hundred milliseconds, but they slowly decay, due to the transition into a desensitized state. This behavior is consistent with long periods of closures separating bursts of channel openings upon prolonged exposure to saturating concentrations of the ligand observed in single channel traces. Within the bursts, the open probability of the channel is high, which is indicative for a high efficacy of the investigated ligands [32],. The detailed kinetic characterization of gating based on single channel recordings will be the subject of future investigations.

It is currently still unclear whether the crystal structure of ELIC, which shows a non-conducting conformation of the channel obtained in the absence of ligands, is closer to the resting or the desensitized state of the channel. Consequently, its relationship to the resting state of eukaryotic receptors is also unknown [19],[47]. A previous hypothesis that the observed conformation would be induced by strong interactions of aromatic phenylalanine residues at the extracellular pore entry [31], however, could not be substantiated since the mutation to alanine did not change the overall structure and instead stabilized the closed state of the channel as indicated by a decrease in the potency of the agonist. This observation is unexpected, as hydrophobic interactions generally play an important role in the stabilization of protein structures.

In an allosteric model of channel activation, the binding of the ligand shifts the equilibrium between the open and closed channel towards the open state [48],[49]. For that purpose, the agonist has to bind with higher affinity to the open state of the channel. In this respect, it is interesting to compare the conformation of the ligand binding domain of ELIC with a hypothetical conformation of the domain in the open state that was generated by a rigid body transformation to the structure of GLIC (Figure 6B). In this hypothetical conformation, the binding site has contracted and is no longer accessible to the solvent. While the site is still large enough to accommodate the different ligands, their interaction with the protein would likely be stronger, thus fulfilling an important requirement for ligand activation.

With respect to its ion conduction properties, ELIC is closely related to nAChRs, serotonin receptors, and GLIC [13],[34],[37],[50],[51]. ELIC is a cation selective channel that is essentially impermeable for anions. It does not discriminate between different monovalent cations and allows the permeation of divalent cations, which appear to interact stronger with the protein. The single channel conductance of ELIC is high; it is about 10 times higher than observed in GLIC [13], and it is comparable to the fastest conducting members of the family [39],[52],[53]. While a detailed understanding of this property will require more experimental data, it is still interesting to compare the pore geometry of GLIC and a hypothetic open state of ELIC that is based on the conformation of the pore forming helix observed in GLIC (Figure 6B). Despite the similarity of the protein backbone, the pore geometries of ELIC and GLIC would differ in such a model due to the different size of the side-chains of pore-lining residues. While the bulky Phe246 and Leu239 at the extracellular half of the pore of ELIC would result in a smaller pore diameter, the constricting intracellular diameter, which might be the main determinant for the conductance, could be larger (Figure 6B). Part of this larger pore diameter is caused by the location of the conserved Glu229 at the intracellular pore entry that is shifted by one residue in GLIC.

With known agonists, ELIC has now become amenable for detailed structural and functional investigations. A fundamental question of ionotropic receptors concerns the mechanisms by which the binding of ligands induces channel activation over a large distance in a different part of the protein. ELIC is well suited to study the details of this process: Its large conductance will allow investigations of ion conductance and gating on a single channel level, which will additionally be facilitated by its comparably simple functional properties. Its high expression level and biochemical stability make it a suitable candidate for spectroscopy, and finally, knowledge of the ligand opens up the possibility to obtain structural information of a conducting conformation of the protein.

## Materials and Methods

### Protein Expression and Purification

ELIC and the mutants F246A and E229A were expressed and purified as described [10]. *E. coli* BL21DE3 containing a vector encoding for a fusion protein consisting of the pelB signal sequence, a His_10_ tag, maltose binding protein, a HRV 3C protease site, and ELIC were grown in M9 minimal medium at 37°C to an OD of 1.0 and subsequently cooled to 20°C. Expression was induced by addition of 0.3 mM IPTG overnight. All the following steps were carried out at 4°C. The protein was extracted from isolated membranes in a buffer containing 1% n-Undecyl-β-D-Maltoside (UDM, Anatrace, Inc.) and purified by Ni-NTA chromatography (Qiagen). The purified MBP-ELIC-fusion protein was digested with HRV 3C protease to cleave the His_10_-MBP protein. His_10_-MBP and 3C were subsequently removed from solution by binding to Ni-NTA resin. ELIC was concentrated and subjected to gel filtration on a Superdex 200 column (GE Healthcare). The protein peak corresponding to the ELIC pentamer was pooled and concentrated to 10 mg/ml and used for crystallization. For reconstitution into liposomes, an additional anion exchange purification step (POROS HQ, Applied Biosystems) was introduced prior to gel filtration.

### Crystallization and Structure Determination

The purified protein was crystallized in sitting drops at 4°C. Protein containing additional 0.5 mg/ml *E. coli* polar lipids (Avanti Polar Lipids, Inc.) was mixed in a 1∶1 ratio with reservoir solution (100 mM ADA pH 6.5, 200 mM NaLiSO_4_, and 10% (w/v) PEG4000). Bromopropylamine was soaked into the crystals by incubation with solutions containing 5 mM of the ligand. The crystals were cryoprotected by transfer into solutions containing 30% glycerol. All datasets were collected on frozen crystals on the X06SA beamline at the Swiss Light Source (SLS) of the Paul Scherrer Institut (PSI) on a PILATUS detector (Dectris). The data were indexed, integrated, and scaled with XDS [54] and further processed with CCP4 programs [55]. The structure of the F246A mutant at 3.3 Å was determined by Molecular Replacement using the structure of ELIC as the search model. The model was rebuilt in O [56] and refined maintaining strong 10-fold NCS constraints in PHENIX [57]. The structures of the WT protein in complex with bromopropylamine at 4.0 Å was improved by rigid body refinement in CNS [58]. R and R_free_ were monitored throughout. R_free_ was calculated by selecting 5% of the reflection data in thin slices that were selected for the initial dataset of ELIC and that were omitted in refinement (Table 3).

### Reconstitution and Planar Lipid Bilayer Experiments

ELIC was reconstituted into *E. coli* polar lipids that were solubilized in reconstitution buffer (450 mM KCl, 25 mM citric acid, 25 mM phosphoric acid, pH 7.0) containing 35 mM CHAPS. The protein was added at a protein-to-lipid ratio of 1 to 10 µg/mg with a final lipid concentration of 15 mg/ml. The detergent was removed by dialysis, and liposomes were frozen in liquid nitrogen and stored at −80°C. Liposomes containing ELIC were fused to bilayers formed from POPE/POPG lipids (in molar ratio of 1∶3, Avanti) and recorded in a horizontal planar lipid bilayer system as previously described [59]. Electrodes were connected to the respective bath solutions via salt bridges. Solutions were prepared using 10 mM HEPES as buffer. NaCl and NaOH were added to reach desired Na^+^ concentration. Solutions containing K^+^ ions were prepared in a similar way but using KOH and KCl instead. Cs^+^ solutions were prepared in 10 mM Tris. For all solutions pH was adjusted to 7.0 with HCl. Low salt solutions contained equivalent concentrations of mannitol to correct the osmotic pressure. Currents were recorded with an Axopatch amplifier 200B (Axon Instruments, Inc.). Data were sampled at 100 µs, filtered at 1,000 Hz, and analyzed using Clampfit (Axon Instruments, Inc.).

### Two Electrode Voltage Clamp Recording

Constructs containing the gene of either the WT channel or point mutants preceded by the signal sequence of the chicken α7nAchR were cloned into the pTLN vector for expression in *X. laevis* oocytes [60]. After linearization of the plasmid DNA by MluI, capped complementary RNA was transcribed with the mMessage mMachine kit (Ambion) and purified with the RNeasy kit (Qiagen). For expression, 1–50 ng of RNA was injected into defolliculated oocytes. Two-electrode voltage clamp measurements were performed one day after injection at 20°C (OC-725B, Warner Instrument Corp.). Currents were recorded in bath solutions containing 10 mM HEPES (pH 7), 130 mM NaCl, and the respective ligands. Cysteamine containing solutions were supplemented with 1 mM DTT.

### Patch Clamp Recording

Membrane patches of *X. laevis* oocytes were recorded in the excised outside-out configuration 2–3 d after injection of mRNA with an Axopatch 200B amplifier (Axon Instruments) at 20°C. Data were sampled at 100 µs, filtered with 1,000 Hz, and analyzed using Clampfit (Axon Instruments, Inc.). Bath solutions contained 10 mM HEPES (pH 7.0), 150 mM NaCl, 0.5 mM BaCl_2_, and the respective ligands. The electrodes had a resistance of 2–3 MΩ. Pipette solutions contained 150 mM NaCl, 1 mM EGTA, 5 mM MgCl, and 10 mM HEPES at pH 7.0. Bath electrodes were placed in 1 M KCl solution connected to the bath solution by Agar bridges. The agonists were applied to the patch using a stepper motor (SF77B Perfusion fast step, Warner).

### Accession Code

The coordinates of the ELIC mutant F246A have been deposited with the Protein Data Bank under code 2yks.

## Supporting Information

Figure S1 Pore geometries of pentameric ligand-gated ion channels. (A) ELIC and GLIC pentamers were superimposed by a least square fit of the α1 helices. The Cα traces of single subunits of the superimposed structures (ELIC, orange; GLIC, green) are shown. The structures are viewed from within the membrane. The rotation axes describing the main movements in the extracellular and pore domains are indicated. (B) View of the superimposed α2–α3 helix pairs of a single subunit. The color coding is as in (A). The rotation axis is indicated. (C) View of the superimposed α2–α3 helix pairs of the ELIC and GLIC pentamers from within the membrane. The front subunit is removed for clarity. (D) View of the superimposed α2–α3 helix pairs of ELIC and the structure of the nAChR, which was determined by electron crystallography at 4.0 Å resolution. (E) View of the α2 helices of ELIC defining the pore region. The front subunit is removed for clarity. The molecular surface is shown as white mesh. (E) View of the α2 helices of GLIC defining the pore region. The front subunit is removed for clarity. The molecular surface is shown as white mesh (G) Sequence alignment of the pore regions of ELIC, GLIC, the human α7 nicotinic acetylcholine receptor (nAChR), and the α3 subunit of the human GABA-receptor (GABAR). Conserved residues of cation selective channels that are lining the pore region are highlighted (yellow, hydrophobic; green, hydrophilic; red, negatively charged). A conserved proline residue in the α2–α3 linker is highlighted in grey.
(TIF)

Figure S2 Comparison of the ligand binding domains of ELIC and the AChBP. (A) Cα trace of the AChBP. The regions A–F that constitute the ligand-binding site are shown in unique colors and labeled. The secondary structure elements are indicated. (B) Stereo view of the AChBP superimposed on the ligand binding domain of ELIC and the AChBP. The proteins are shown as Cα traces. The principle and complementary sides are shown in green and orange, respectively. The light colors correspond to the AChBP. (C) Ligand binding site of the AChBP. Bound carbamylcholine is shown in magenta. Selected residues of the binding pocket and the secondary structure elements are labeled. (D) Ligand binding site of ELIC. Selected residues of the binding pocket and the secondary structure elements are labeled. (E) Stereo view of the superimposed structures of the binding regions of ELIC and the AChBP.
(TIF)

Figure S3 Library of potential agonists. (A) List of molecules used for screening of agonists. (B) Current response of ELIC to isobutylamine and putrescine at pH 7.0. Currents were recorded from *Xenopus laevis* oocytes at −60 mV with the two electrode voltage clamp technique. Traces show the response to agonists in comparison to the response to cysteamine. Agonists were applied to the outside in a concentration of 10 mM. The activation by cysteamine (grey bar, 2 mM) is followed by the application of the respective agonist (black bar, *, 10 mM) and another activation by cysteamine (grey bar, 2 mM).
(TIF)

Figure S4 Activation, deactivation, and desensitization of ELIC. (A) Decay of currents in two-electrode voltage clamp recordings. *Xenopus laevis* oocytes expressing ELIC were incubated in solutions containing 5 mM cysteamine for an extended amount of time (black bar). The voltage was clamped to the reversal potential and the fraction of open channels was assayed by brief steps to −60 mV. (B) Activation and desensitization of macroscopic currents measured from excised outside-out patches. Solutions containing either no ligand or 5 mM cysteamine (black bar) were applied by fast solution exchange. The currents decay over time in the presence of ligand, but full channel activity was recovered after washout and incubation of the patch in the absence of ligand. (C) Activation and deactivation kinetics obtained from macroscopic currents measured from outside-out patches in response to application and washout of cysteamine. Solutions were exchanged using a stepper motor (SF77B Perfusion fast step, Warner). Currents were fitted to a single exponential function. The activation time constant decreased with increasing ligand concentration as expected for a ligand-gated channel. Averages from nine independent measurements are shown. The deactivation time constant was independent of the ligand concentration. Averages of 27 independent measurements are shown. All errors are standard deviations (s.d.).
(TIF)

Figure S5 Conservation of the ligand-binding site. (A) Sequence alignment of sections of the extracellular domain constituting the ligand-binding site. Strongly conserved residues are colored in red. Residues lining the ligand-binding site are highlighted (green, aromatic residues conserved in all pLGICs; red, positions conserved in ELIC, GABA, and glycine receptors; yellow, residues in ELIC that are not conserved). (B) Structure of the ligand-binding region of ELIC. The conservation of residues is indicated.
(TIF)

Figure S6 Single channel analysis. (A) Single channel trace of ELIC recorded in planar lipid bilayers. The voltage was clamped to 100 mV. Channel activity was induced by addition of 2 mM cysteamine. The trace shows the occasional transition of the open channel to a subconductance of about 80% of the maximum current amplitude. (B) Single channel traces of ELIC expressed in *Xenopus laevis* oocytes recorded in excised outside-out patches (left) and of reconstituted protein recorded in planar lipid bilayers (right). The current histograms of the respective traces and a fit to a Gaussian are shown. (C) Current-voltage relationships in asymmetric salt solutions. The “extracellular side” contained 150 mM NaCl, and the “intracellular side” contained 150 mM of either CaCl_2_ or BaCl_2_. Channels were activated by addition of 2 mM cysteamine to the “extracellular side.” (D) Current-voltage relationships of the mutant E229A in symmetric concentrations of either 300 mM NaCl or 300 mM Na-glucuronate (NaGlc). Channels were activated by addition of 2 mM cysteamine to the “extracellular side.” Data from the WT channel are shown for comparison. Data shown in panels C and D were measured from single channels in planar lipid bilayers. The errors are s.d. obtained from fits to current amplitude histograms.
(TIF)

## Abbreviations

AChBP: acetylcholine binding protein
nAChRs: nicotinic acetylcholine receptors
pLGICs: pentameric ligand-gated ion channels
