# Supplementary figures and images for: Ligand Activation of the Prokaryotic Pentameric Ligand-Gated Ion Channel ELIC
Source: PLoS Biol. 2011 Jun 21;9(6):e1001101. doi: 10.1371/journal.pbio.1001101 (PMC3119659; doi:10.1371/journal.pbio.1001101)

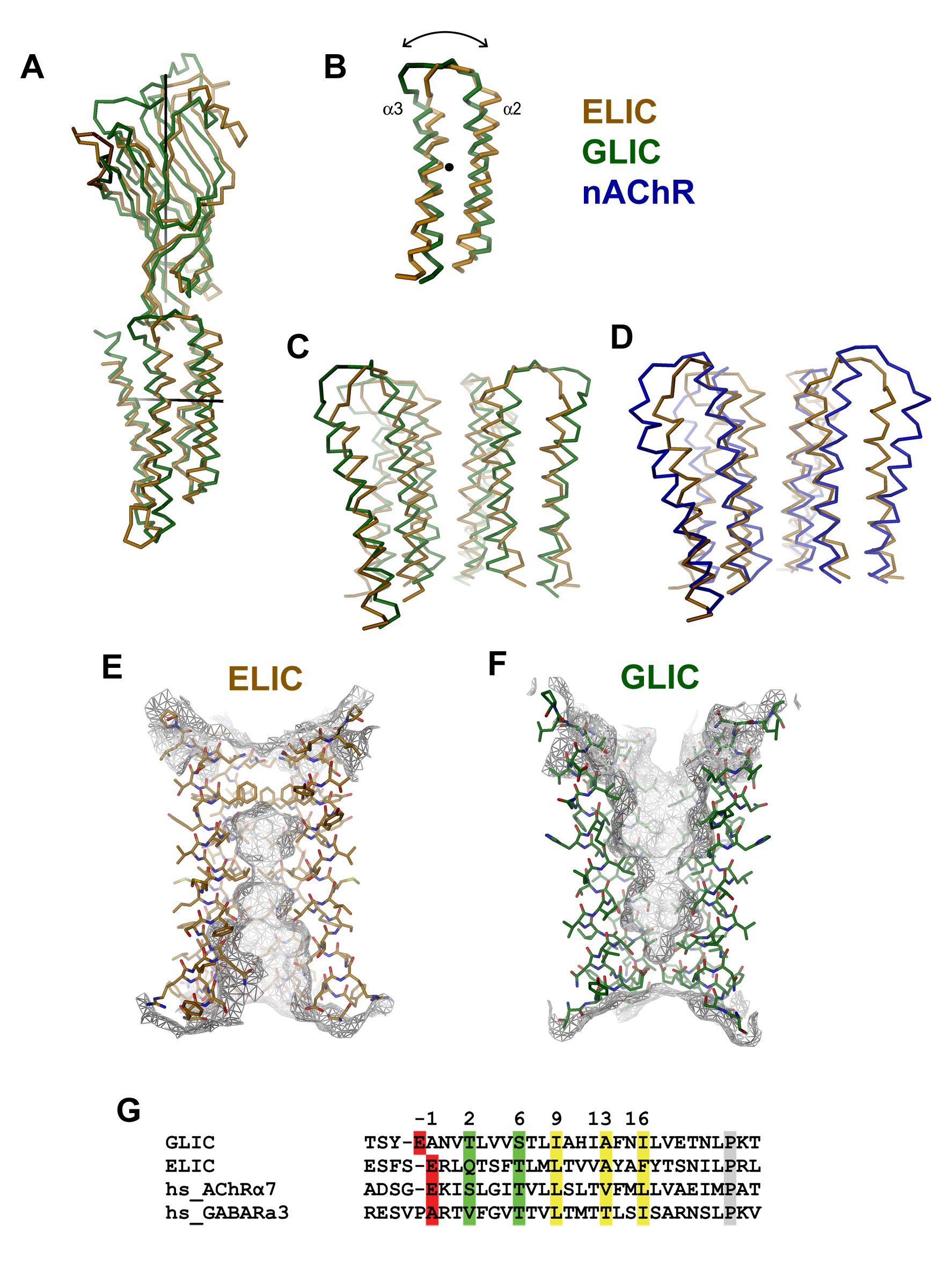

Supplement: Figure S1 — Pore geometries of pentameric ligand-gated ion channels. (A) ELIC and GLIC pentamers were superimposed by a least square fit of the α1 helices. The Cα traces of single subunits of the superimposed structures (ELIC, orange; GLIC, green) are shown. The structures are viewed from within the membrane. The rotation axes describing the main movements in the extracellular and pore domains are indicated. (B) View of the superimposed α2–α3 helix pairs of a single subunit. The color coding is as in (A). The rotation axis is indicated. (C) View of the superimposed α2–α3 helix pairs of the ELIC and GLIC pentamers from within the membrane. The front subunit is removed for clarity. (D) View of the superimposed α2–α3 helix pairs of ELIC and the structure of the nAChR, which was determined by electron crystallography at 4.0 Å resolution. (E) View of the α2 helices of ELIC defining the pore region. The front subunit is removed for clarity. The molecular surface is shown as white mesh. (E) View of the α2 helices of GLIC defining the pore region. The front subunit is removed for clarity. The molecular surface is shown as white mesh (G) Sequence alignment of the pore regions of ELIC, GLIC, the human α7 nicotinic acetylcholine receptor (nAChR), and the α3 subunit of the human GABA-receptor (GABAR). Conserved residues of cation selective channels that are lining the pore region are highlighted (yellow, hydrophobic; green, hydrophilic; red, negatively charged). A conserved proline residue in the α2–α3 linker is highlighted in grey. (TIF) [file pbio.1001101.s001.tif]

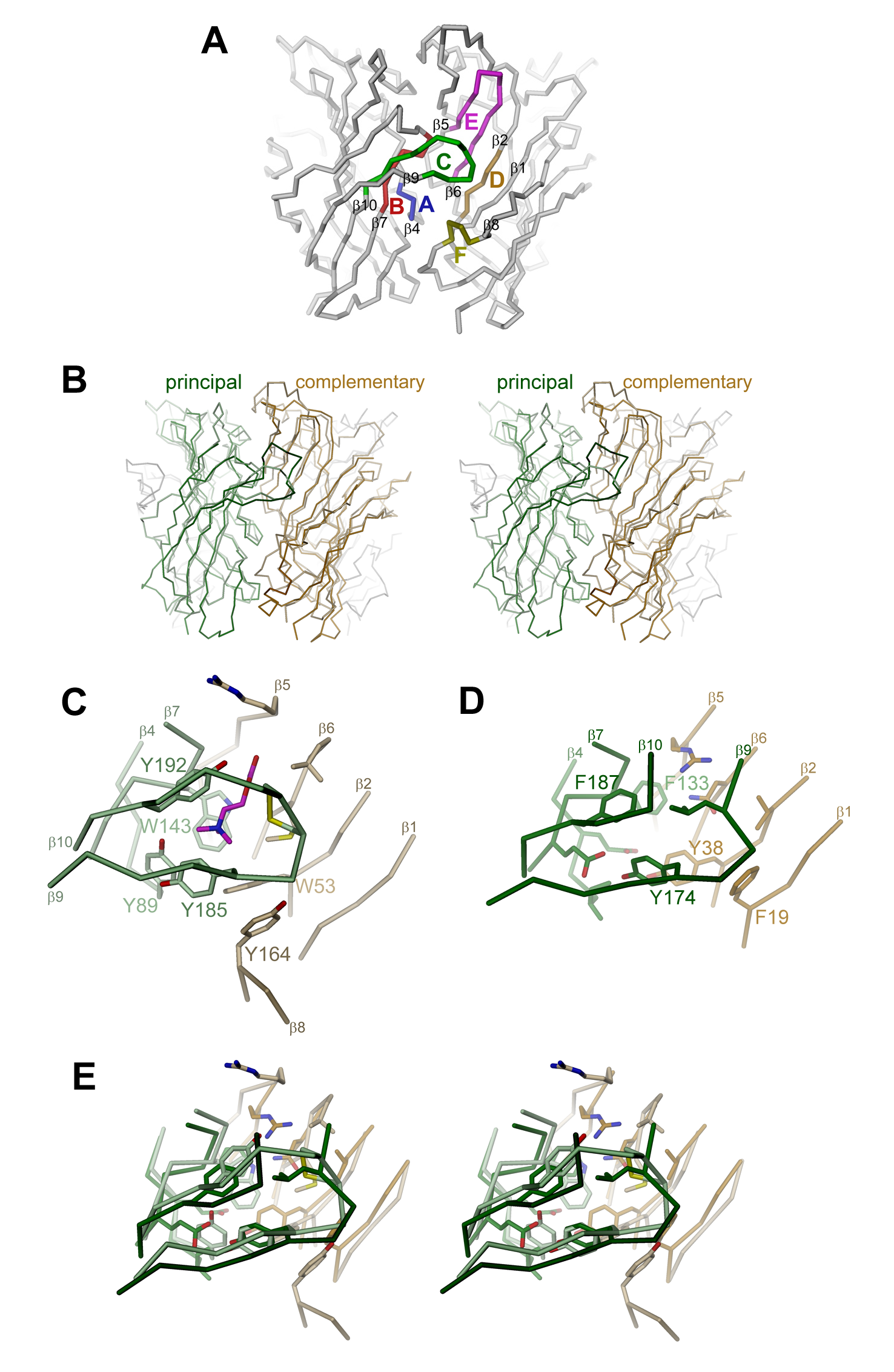

Supplement: Figure S2 — Comparison of the ligand binding domains of ELIC and the AChBP. (A) Cα trace of the AChBP. The regions A–F that constitute the ligand-binding site are shown in unique colors and labeled. The secondary structure elements are indicated. (B) Stereo view of the AChBP superimposed on the ligand binding domain of ELIC and the AChBP. The proteins are shown as Cα traces. The principle and complementary sides are shown in green and orange, respectively. The light colors correspond to the AChBP. (C) Ligand binding site of the AChBP. Bound carbamylcholine is shown in magenta. Selected residues of the binding pocket and the secondary structure elements are labeled. (D) Ligand binding site of ELIC. Selected residues of the binding pocket and the secondary structure elements are labeled. (E) Stereo view of the superimposed structures of the binding regions of ELIC and the AChBP. (TIF) [file pbio.1001101.s002.tif]

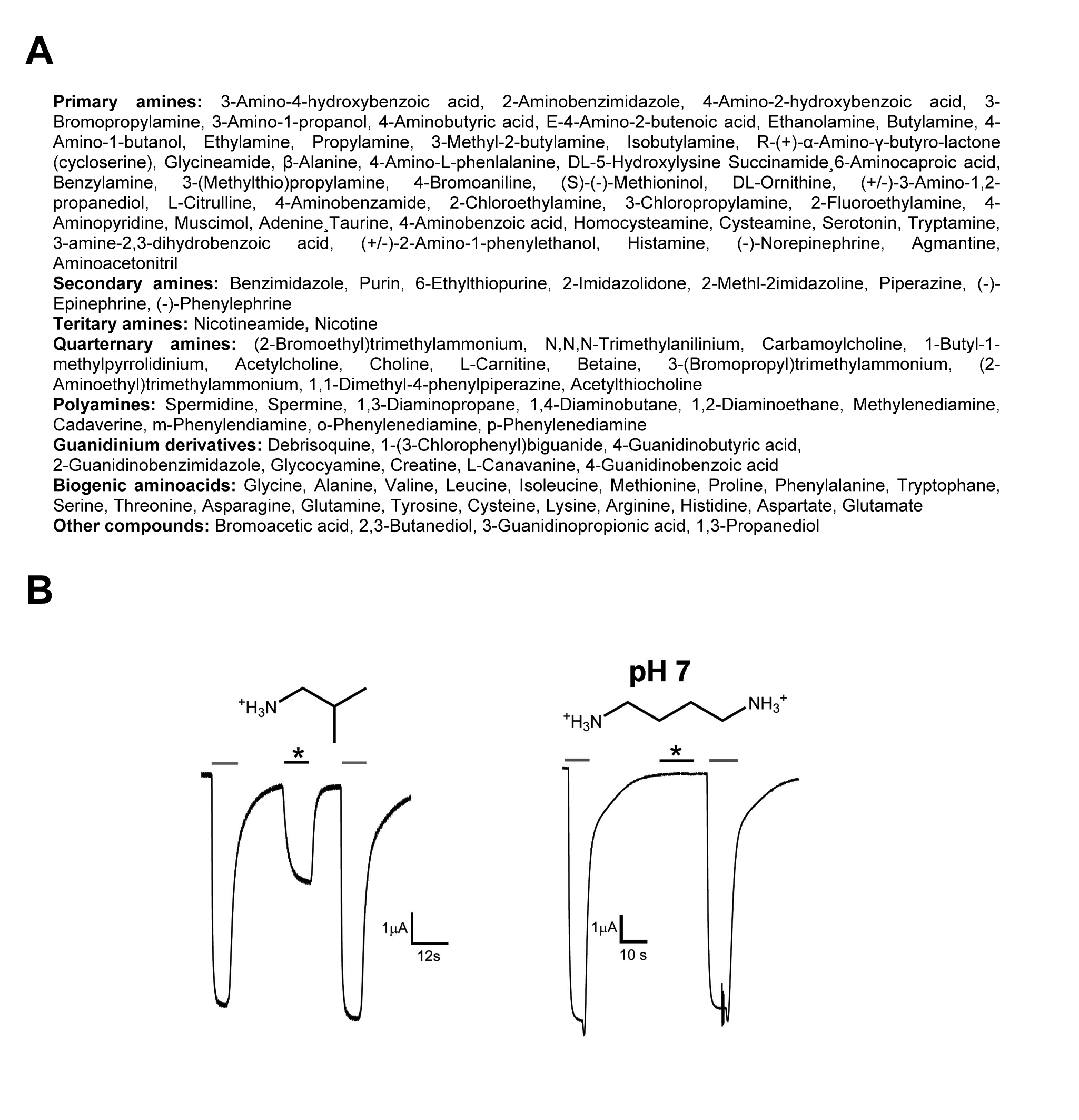

Supplement: Figure S3 — Library of potential agonists. (A) List of molecules used for screening of agonists. (B) Current response of ELIC to isobutylamine and putrescine at pH 7.0. Currents were recorded from Xenopus laevis oocytes at −60 mV with the two electrode voltage clamp technique. Traces show the response to agonists in comparison to the response to cysteamine. Agonists were applied to the outside in a concentration of 10 mM. The activation by cysteamine (grey bar, 2 mM) is followed by the application of the respective agonist (black bar, *, 10 mM) and another activation by cysteamine (grey bar, 2 mM). (TIF) [file pbio.1001101.s003.tif]

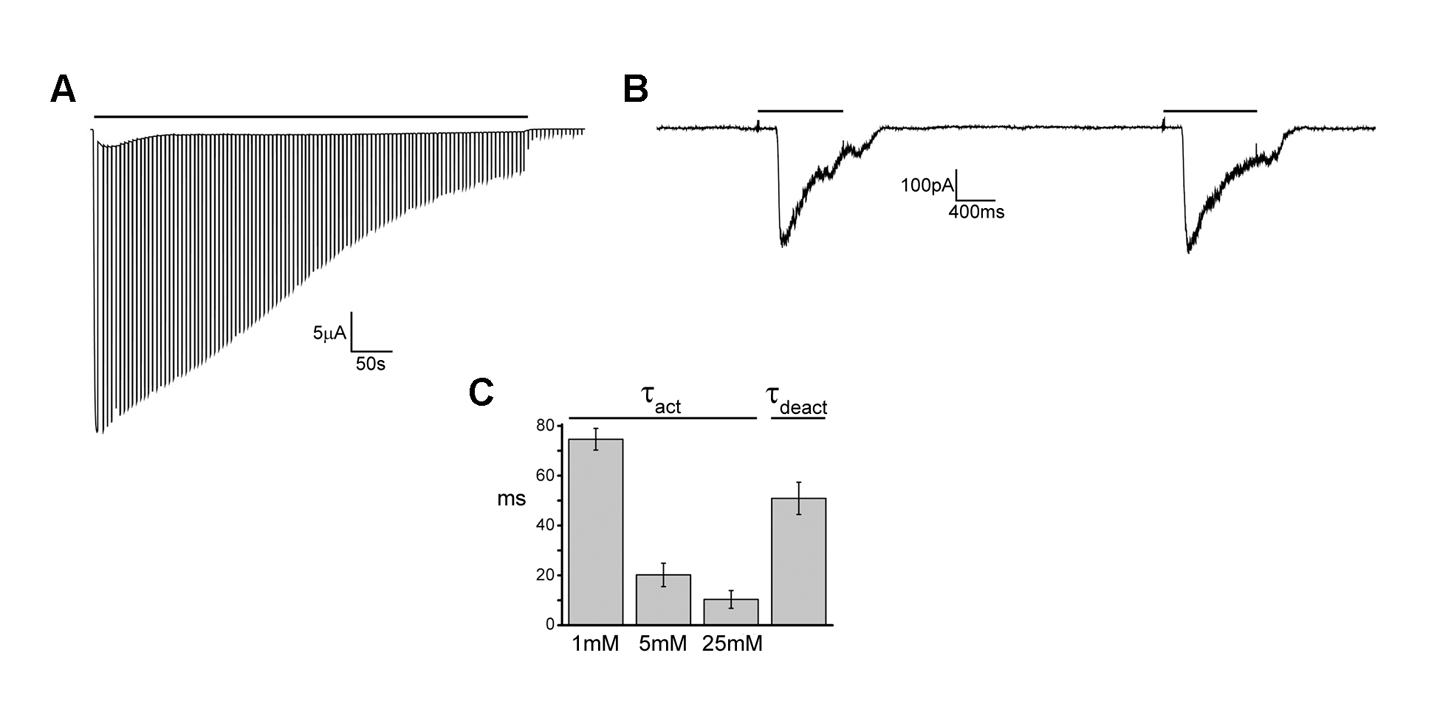

Supplement: Figure S4 — Activation, deactivation, and desensitization of ELIC. (A) Decay of currents in two-electrode voltage clamp recordings. Xenopus laevis oocytes expressing ELIC were incubated in solutions containing 5 mM cysteamine for an extended amount of time (black bar). The voltage was clamped to the reversal potential and the fraction of open channels was assayed by brief steps to −60 mV. (B) Activation and desensitization of macroscopic currents measured from excised outside-out patches. Solutions containing either no ligand or 5 mM cysteamine (black bar) were applied by fast solution exchange. The currents decay over time in the presence of ligand, but full channel activity was recovered after washout and incubation of the patch in the absence of ligand. (C) Activation and deactivation kinetics obtained from macroscopic currents measured from outside-out patches in response to application and washout of cysteamine. Solutions were exchanged using a stepper motor (SF77B Perfusion fast step, Warner). Currents were fitted to a single exponential function. The activation time constant decreased with increasing ligand concentration as expected for a ligand-gated channel. Averages from nine independent measurements are shown. The deactivation time constant was independent of the ligand concentration. Averages of 27 independent measurements are shown. All errors are standard deviations (s.d.). (TIF) [file pbio.1001101.s004.tif]

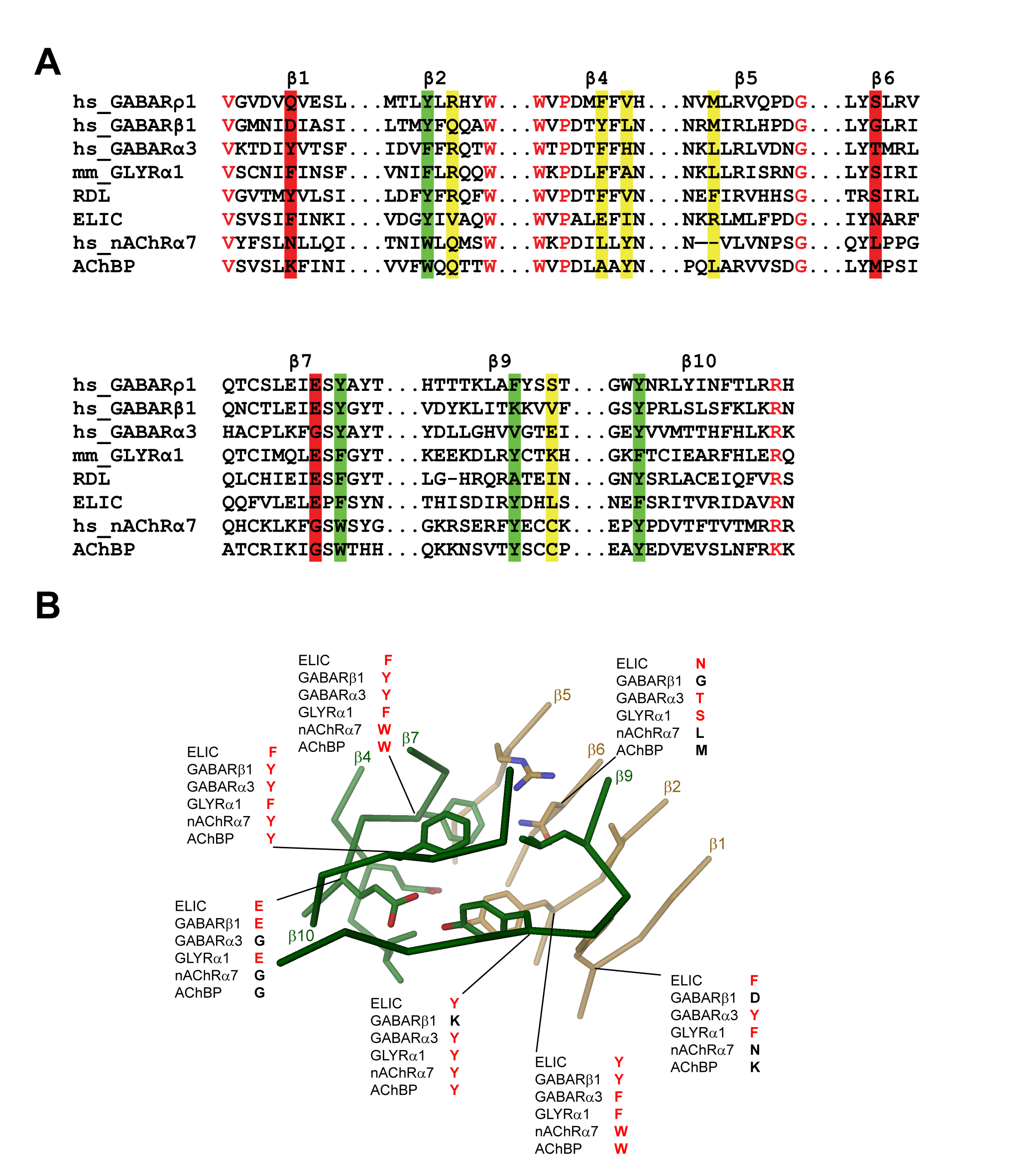

Supplement: Figure S5 — Conservation of the ligand-binding site. (A) Sequence alignment of sections of the extracellular domain constituting the ligand-binding site. Strongly conserved residues are colored in red. Residues lining the ligand-binding site are highlighted (green, aromatic residues conserved in all pLGICs; red, positions conserved in ELIC, GABA, and glycine receptors; yellow, residues in ELIC that are not conserved). (B) Structure of the ligand-binding region of ELIC. The conservation of residues is indicated. (TIF) [file pbio.1001101.s005.tif]

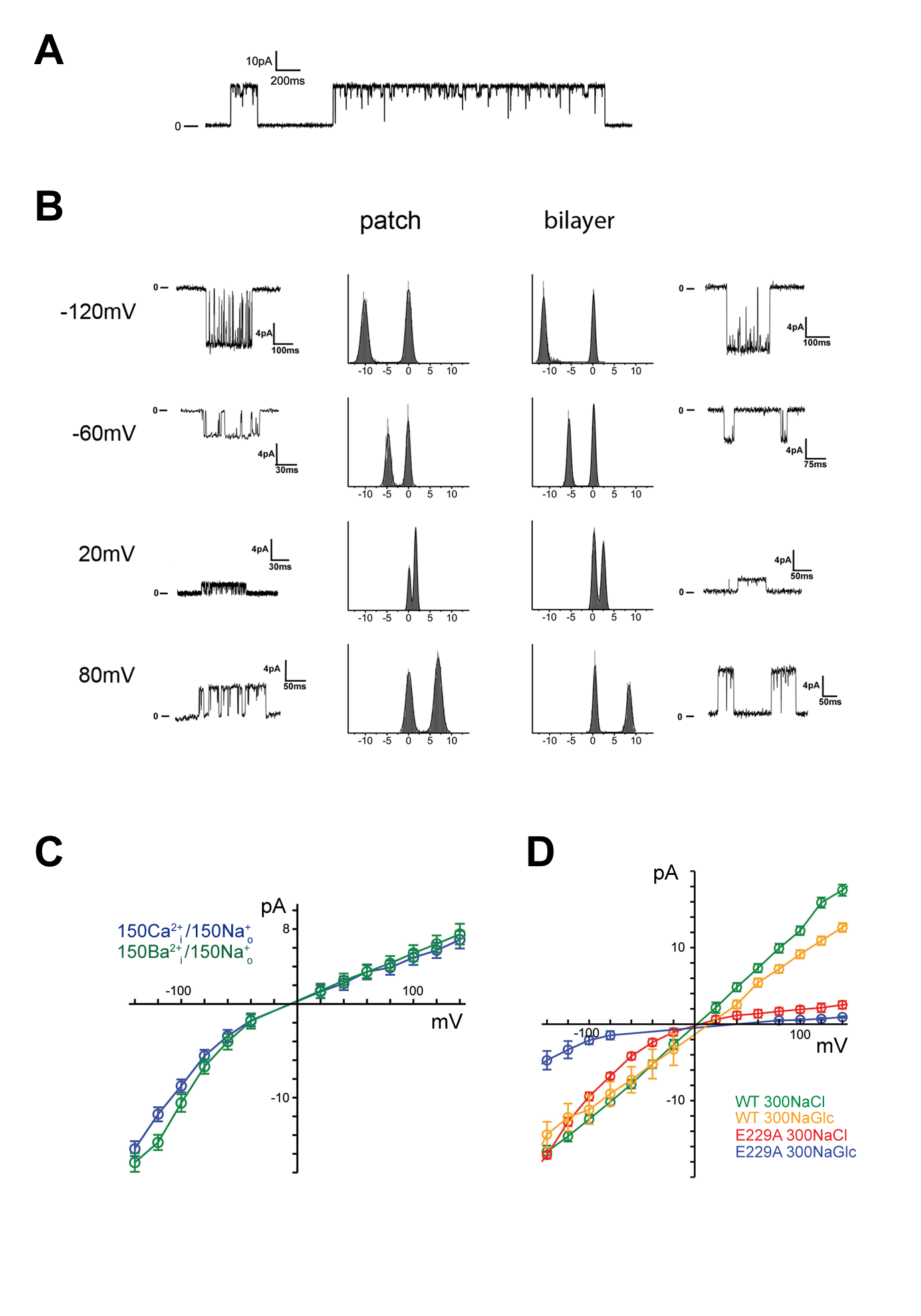

Supplement: Figure S6 — Single channel analysis. (A) Single channel trace of ELIC recorded in planar lipid bilayers. The voltage was clamped to 100 mV. Channel activity was induced by addition of 2 mM cysteamine. The trace shows the occasional transition of the open channel to a subconductance of about 80% of the maximum current amplitude. (B) Single channel traces of ELIC expressed in Xenopus laevis oocytes recorded in excised outside-out patches (left) and of reconstituted protein recorded in planar lipid bilayers (right). The current histograms of the respective traces and a fit to a Gaussian are shown. (C) Current-voltage relationships in asymmetric salt solutions. The “extracellular side” contained 150 mM NaCl, and the “intracellular side” contained 150 mM of either CaCl2 or BaCl2. Channels were activated by addition of 2 mM cysteamine to the “extracellular side.” (D) Current-voltage relationships of the mutant E229A in symmetric concentrations of either 300 mM NaCl or 300 mM Na-glucuronate (NaGlc). Channels were activated by addition of 2 mM cysteamine to the “extracellular side.” Data from the WT channel are shown for comparison. Data shown in panels C and D were measured from single channels in planar lipid bilayers. The errors are s.d. obtained from fits to current amplitude histograms. (TIF) [file pbio.1001101.s006.tif]
